# Initiation of human colon cancer cell proliferation by trypsin acting at protease-activated receptor-2

**DOI:** 10.1054/bjoc.2001.1976

**Published:** 2001-09

**Authors:** D Darmoul, J-C Marie, H Devaud, V Gratio, M Laburthe

**Affiliations:** Neuroendocrinologie et Biologie Cellulaire Digestives, Institut National de la Santé et de la Recherche Médicale, INSERM U410, Faculté de Médecine Xavier Bichât, Paris, 75018, France

**Keywords:** colon cancer, proliferation, protease-activated receptor-2, trypsin, serine protease

## Abstract

The protease-activated receptor-2 (PAR-2) is a G protein-coupled receptor that is cleaved and activated by trypsin. We investigated the expression of PAR-2 and the role of trypsin in cell proliferation in human colon cancer cell lines. A total of 10 cell lines were tested for expression of PAR-2 mRNA by Northern blot and RT-PCR. PAR-2 protein was detected by immunofluorescence. Trypsin and the peptide agonist SLIGKV (AP2) were tested for their ability to induce calcium mobilization and to promote cell proliferation on serum-deprived cells. PAR-2 mRNA was detected by Northern blot analysis in 6 out of 10 cell lines [HT-29, Cl.19A, Caco-2, SW480, HCT-8 and T84]. Other cell lines expressed low levels of transcripts, which were detected only by RT-PCR. Further results were obtained with HT-29 cells: (1) PAR-2 protein is expressed at the cell surface; (2) an increase in intracellular calcium concentration was observed upon trypsin (1–100 nM) or AP2 (10–100 μM) challenges; (3) cells grown in serum-deprived media supplemented with trypsin (0.1–1 nM) or AP2 (1–300 μM) exhibited important mitogenic responses (3-fold increase of cell number). Proliferative effects of trypsin or AP2 were also observed in other cell lines expressing PAR-2. These data show that subnanomolar concentrations of trypsin, acting at PAR-2, promoted the proliferation of human colon cancer cells. The results of this study indicate that trypsin could be considered as a growth factor and unravel a new mechanism whereby serine proteases control colon tumours. © 2001 Cancer Research Campaign http://www.bjcancer.com


British Journal of Cancer (2001) **85**(5), 772–779
© 2001 Cancer Research Campaign
doi: 10.1054/ bjoc.2001.1976, available online at http://www.idealibrary.com on IDEAL

http://www.bjcancer.com

### Initiation of human colon cancer cell proliferation by trypsin acting at protease-activated receptor-2

**D Darmoul, J-C Marie, H Devaud, V Gratio and M Laburthe**

Neuroendocrinologie et Biologie Cellulaire Digestives, INSERM U410, Institut National de la Santé et de la Recherche Médicale, Faculté de Médecine Xavier Bichât, 75018 Paris, France

**Summary** The protease-activated receptor-2 (PAR-2) is a G protein-coupled receptor that is cleaved and activated by trypsin. We investigated the expression of PAR-2 and the role of trypsin in cell proliferation in human colon cancer cell lines. A total of 10 cell lines were tested for expression of PAR-2 mRNA by Northern blot and RT-PCR. PAR-2 protein was detected by immunofluorescence. Trypsin and the peptide agonist SLIGKV (AP2) were tested for their ability to induce calcium mobilization and to promote cell proliferation on serum-deprived cells. PAR-2 mRNA was detected by Northern blot analysis in 6 out of 10 cell lines [HT-29, Cl.19A, Caco-2, SW480, HCT-8 and T84]. Other cell lines expressed low levels of transcripts, which were detected only by RT-PCR. Further results were obtained with HT-29 cells: (1) PAR-2 protein is expressed at the cell surface; (2) an increase in intracellular calcium concentration was observed upon trypsin (1–100 nM) or AP2 (10–100 μM) challenges; (3) cells grown in serum-deprived media supplemented with trypsin (0.1–1 nM) or AP2 (1–300 μM) exhibited important mitogenic responses (3-fold increase of cell number). Proliferative effects of trypsin or AP2 were also observed in other cell lines expressing PAR-2. These data show that subnanomolar concentrations of trypsin, acting at PAR-2, promoted the proliferation of human colon cancer cells. The results of this study indicate that trypsin could be considered as a growth factor and unravel a new mechanism whereby serine proteases control colon tumours. © 2001 Cancer Research Compaign http://www.bjcancer.com

**Keywords**: colon cancer, proliferation, protease-activated receptor-2, trypsin, serine protease

Proteolytic enzymes such as matrix metalloproteases (MMPs) and various serine proteases are known to mediate cancer progression and metastasis (Mignatti and Rifkin, 1993; Sorsa et al, 1997; Mazzieri et al, 1997; Blasi and Stoppelli, 1999; Ramos-DeSimone et al, 1999; McCawley and Matrisian, 2000). The importance of trypsin, a major serine protease, has been evidenced more recently in many cancers including tumours in the digestive tract:

1. Extra-pancreatic production of trypsin was shown in ovarian (Hirahara et al, 1995), lung (Kawano et al, 1997), gastric (Miyata et al, 1998) and colonic tumours (Bernard-Perrone et al, 1998; Miyata et al, 1999);
2. The trypsinogen gene is significantly expressed in vascular endothelial cells around gastric tumours (Koshikawa et al, 1997);
3. Elevated serum trypsin level has been reported in some digestive cancers (Hedstrom et al, 1996; Ichikawa et al, 2000);
4. Overexpression of exogenous trypsinogen cDNA in human gastric cancer cells increases their tumourigenicity in nude mice (Miyata et al, 1998);
5. Serine protease inhibitors suppress carcinogenesis in many different in vivo and in vitro assay systems (Kennedy, 1994; Kennedy, 1998).

Although trypsin, like other proteolytic enzymes, contributes to cancer progression by degradation of extracellular matrix proteins (Koshikawa et al, 1992), a direct effect on tumour cell proliferation is conceivable. Indeed, it has long been known that trypsin stimulates cell division of fibroblasts by acting at the cell surface (Blumberg and Robbins, 1975; Carney and Cunningham, 1977). This is in line with recent studies suggesting that the action of trypsin can be mediated not only by its classical ability to catalyze the hydrolysis of various proteins, but also through specific cleavage and activation of a cell surface receptor, the protease-activated receptor-2 (PAR-2) (Nystedt et al, 1994; Bohm et al, 1996b; Hollenberg, 1996).

PAR-2 is the second member of a new subfamily of G protein-coupled receptors activated by proteolytic cleavage (Dery et al, 1998) that also includes PAR-1, PAR-3 and PAR-4 (Vu et al, 1991; Ishihara et al, 1997; Xu et al, 1998), which are receptors for thrombin. PAR-2 cleavage by trypsin exposes a new amino terminus peptide that functions as tethered ligand (Nystedt et al, 1994; Bohm et al, 1996b). This new ligand binds to the core of the receptor and initiates signal transduction resulting in stimulation of phosphoinositide breakdown and cytosolic calcium mobilization (Nystedt et al, 1994; Dery et al, 1998). A short synthetic peptide (activating peptide-2 or AP2) corresponding to the new amino-terminus exposed after trypsin cleavage, is able to activate selectively the PAR-2 receptor and mimic the cellular effects of trypsin (Nystedt et al, 1994, 1995; Saifeddine et al, 1996; Bohm et al, 1996b). Therefore AP2 or AP2-modified sequences (Vergnolle, 2000) have become useful specific pharmacological ligands used to assess the biological function of PAR-2 in several biological systems.

The presence of PAR-2 has been revealed in a variety of tissues (Nystedt et al, 1994; D'Andrea et al, 1998); in particular high expression has been observed in the gastrointestinal tract (Nystedt

*Received 2 March 2001*
*Revised 14 May 2001*
*Accepted 16 May 2001*

*Correspondence to:* M Laburthe

BJOC 01-1976 772-779 20/8/01 3:33 pm Page 773

et al, 1995; Bohm et al, 1996b; Kong et al, 1997; D'Andrea et al, 1998; Nguyen et al, 1999). These receptors are functional since activation of PAR-2 by trypsin or AP2 induces ion secretion and prostaglandin production in normal intestine (Kong et al, 1997; Vergnolle et al, 1998). Despite increasing evidence for a role of PAR-2 in normal digestive epithelia, the expression and functional consequences of activation of PAR-2 in digestive cancers has not yet been reported.

The experiments described in this article were carried out to determine the expression of PAR-2 in human colon cancer cells and to investigate the possible role of trypsin and PAR-2 in colon cancer cell proliferation. We used Northern blot, reverse-transcription polymerase chain reaction (RT-PCR) and immunofluorescence to characterize PAR-2 in a large collection of human colon cancer cell lines. We further demonstrated its functional coupling to the mobilization of intracellular calcium. Finally, we provided evidence for a major role of PAR-2 in the proliferation of colon cancer cells by showing that its natural activator, trypsin, or the synthetic PAR-2-activating peptide SLIGKV promoted a dramatic increase of colon cancer cell number in culture. The effect of trypsin can account for as much as 30% of the effect of fetal calf serum, which reached a maximum of ~10-fold above serum-starved controls. These data provide the first evidence that PAR-2 is prevalent in human colon cancer and that it plays a very significant role in the control of colon cancer cell growth.

### MATERIALS AND METHODS

### Reagents

The activating peptide AP2 (SLIGKV), the reverse sequence RP (VKGILS) and the peptide (SLIGKVDGTSHVTG) were obtained from Neosystem (Strasbourg, France). Trypsin (16 000 u.mg$^{-1}$) and goat anti-mouse IgG coupled to fluorescein were from Sigma chemical Co. (St Louis, MO, USA). Fura-2/AM and pleuronic acid were obtained from Molecular probes (Leiden, Netherlands). Synthetic oligonucleotides, fetal calf serum (FCS), DMEM, RPMI 1640 and HAM F12 were from GIBCO-BRL (Cergy-Pontoise, France). Hybond membrane and radioactive isotope [$^{32}$P] were from Amersham (Les Ullis, France). Human PAR-2 cDNA (Nystedt et al, 1995) was generously provided by Dr Sundelin (Lund University, Lund, Sweden). Glyceraldehyde-3-phosphate dehydrogenase (GAPDH) cDNA was obtained from Clontech (Montigny, France). All other chemicals of the highest purity commercially available were from Bioprobe (Paris, France) or Interchim (Asnière, France).

### Cell lines

The human colon cancer cell lines HT-29, SW480, HCT116, Caco-2, HCT-8, LoVo, LS-174T, SW620 and T84 were obtained from ATCC (Rockville, MD, USA). The Cl.19A cell line was isolated from the parental HT-29 cell line and exhibits enterocyte-like differentiation (Augeron and Laboisse, 1984). The main characteristics of the cell lines used in this study are described elsewhere (Zweibaum et al, 1991). HT-29 (passage 164–170), SW480 (passage 52–63), SW620 (passage 8–12), HCT116 (passage 14–20), Caco-2 (passage 20–23), HCT-8 (passage 20–30), LoVo (passage 4–48), LS-174T (passage 47–55), T84 (passage 63–80) and Cl.19A (passage 13–15) were routinely cultured in 25 cm$^2$ plastic flasks (Costar, Cambridge, MA, USA) as recommended by ATCC. They were maintained at 37˚C in a humidified atmosphere of 5% $CO_2$/air in DMEM containing 4.5 g/l glucose, supplemented with 10% fetal calf serum (FCS) with the exception of Caco-2 cells, which were maintained in 20% FCS and 1% non-essential amino acids.

### Proliferation assay

Determination of cellular proliferation was accomplished by direct cell count. Colon cancer cells were seeded sparsely (5000 cells/well) in 96 cluster wells (Costar) and allowed to attach for 3 d. The culture medium containing 10% FCS was DMEM for HT-29, SW480, HCT116, Caco-2, LS-174T and SW620 cell lines, HAM F12 for LoVo and T84 cell lines and RPMI 1640 for the HCT-8 cell line (Zweibaum et al, 1991). The medium was removed and attached cells were rinsed twice with serum-free medium. They were then grown in 200 µl of culture medium without FCS for 48 h. The cells in representative wells were counted and then 200 µl of a fresh serum-free medium were added with or without trypsin, SLIGKV or VKGILS at different concentrations. A quantity of 10% of FCS was used as a positive control. After different times in culture, cells were detached from triplicate wells by trypsin (0.25% wt/v)–EDTA (0.02% wt/v) and counted in a haemacytometer. Cell death was evaluated with trypan blue. No significant cell death was observed after treatment with AP2, regardless of the concentration, or with trypsin up to 1 nM concentration.

### RNA blot analysis

Total cellular RNA was extracted by acid guanidinium thiocyanate technique (Chirgwin et al, 1979). Samples (10 µg) of total RNA were electrophoresed in 1.5% agarose gels containing 0.6% formaldehyde, blotted onto hybond N Plus membranes and hybridized to a full-length $^{32}$P-labelled PAR-2 cDNA probe overnight at 42˚C in 1 M NaCl, 50% (v/v) formamide, 10% dextran sulfate and 1% SDS. Blots were washed for 20 min with 2X SSC (1X SSC = 150 mM NaCl and 15 mM sodium citrate, pH 7.0), 0.1% SDS at room temperature and then for 30 min with 0.2X SSC, 0.1% SDS at 65˚C. Autoradiograms were exposed at – 70˚C.

### Reverse-transcription polymerase chain reaction (RT-PCR)

A quantity of 4 µg of total RNA were reverse transcribed using oligo (dT) primer. Amplifications were conducted using the same resulting cDNAs. Of the cDNA mixture, 25% was amplified using human PAR-2 sense primer 5′TCC TGC AGT GGC ACC ATC CA3′ and antisense primer 5′TTG CCT TCT TCC TGG AGT GC3′. GAPDH cDNA amplification was used as an internal control with sense primer 5'TCG GAG TCA ACG GAT TTG GTC GTA 3′ and antisense primer 5′AGC CTT CTC CAT GGT GGT GAA GA 3′. Each of the 30 cycles of amplification was performed as follows: 94˚C for 40 s, 50˚C for 40 s and 72˚C for 40 s. PCR products were identified by electrophoresis in 2% agarose gel followed by ethidium bromide staining and blotting by hybridization with $^{32}$P-labelled PAR-2 full length cDNA or $^{32}$P-labelled GADPH cDNA in 5X SSPE, 5X Denhardt's and 0.5% SDS at 65˚C. Blots were washed for 20 min with 2X SSC, 0.1% SDS at room temperature and then twice for 30 min with 0.1X SSC, 0.1% SDS at 65˚C. Autoradiograms were exposed at –70˚C.

*© 2001 Cancer Research Campaign*

BJOC 01-1976 772-779 20/8/01 3:33 pm Page 774

### Immunofluorescence staining

Indirect immunofluorescence was performed on colon cancer cells grown on glass coverslips. The antibodies (SAM11) used in our study were raised against the synthetic peptide SLIGKVDGT-SHVTG, corresponding to residues 37–50 of the human PAR-2 sequence and have been previously characterized (Molino et al, 1997b; 1998). Cells were washed 3 times in PBS and were then incubated with PBS containing 2% BSA for 15 min prior to application of the primary anti-PAR-2 antibody for 2 h at room temperature at a 1:200 dilution. Cells were washed twice for 5 min in PBS containing 1% BSA and secondary antibody, goat anti-mouse IgG coupled to fluorescein, was applied for 45 min at room temperature. The cells were washed again in PBS containing 1% BSA, fixed in 2% paraformaldehyde and finally washed in PBS. The cells were then mounted in glycergel medium (DAKO, High Wycombe, UK). Images were examined on a Leica (Reuil-Malmaison, France) inverted DM IRB microscope using the N2.1 filter (515–560 nm band pass filter).

### Intracellular calcium measurement

Intracellular calcium concentrations of monolayers of colon cancer cells were measured using the fluorescence indicator Fura-2/AM. The cells ($1 \times 10^3$/well) were seeded onto the centre of glass coverslips in cloning cylinders ($0.2\ cm^2 \times 0.8$ cm) and cultured for 3 d. The cells attached to coverslips were then loaded with 5 μM Fura-2/AM in Hepes-buffered saline (135 mM NaCl, 4.6 mM KCl, 1.2 mM $MgCl_2$, 11 mM Hepes, 11 mM glucose, and 1.5 mM $CaCl_2$ at pH 7.4) containing 0.01% pleuronic acid in the dark for 60 min at 37˚C. Then, the cells were washed 3 times with the same buffer and coverslips were placed at the bottom of an open perfusion chamber built for microscope work. The chamber was placed on the stage of an inverted fluorescence microscope (Diaphot, NIKON) and maintained at 37˚C in a climate box. A flow rate of 1 ml/min was used during the whole experiment. The time necessary for a complete change of the medium in the chamber was 140 s. This delay was taken into account in the graphical representations. The microscope was equipped with a × 40 fluor oil-immersion objective. A selected area of cells was excited at 340 nm and 380 nm alternately (every 2 s) and the fluorescence intensity emitted at 510 nm was measured with a PhotoscanII micro-fluorimeter (Photon Technology International, Kontron, France). The measurements of successive 340 nm/380 nm fluorescence ratios reflect the cytosolic concentration of $Ca^{2+}$.

### Statistical analysis

Experiments were performed in triplicate and results are expressed as mean ± SEM. Statistical significance of differences between mean values was assessed with the Student's *t*-test for unpaired data. A *P* value of $< 0.05$ was regarded as statistically significant.

### RESULTS

### Expression of PAR-2 receptor by human colon cancer cell lines

Since no radioactive ligand exists yet for detecting PAR-2, we first investigated the presence of specific PAR-2 mRNA transcripts in total RNA extracted from postconfluent human colon cancer cell lines in culture. Using 10 cell lines exhibiting different phenotypes, a single 3.5 kb transcript was detected in Caco-2, HT-29, T84, Cl.19A, SW480 and HCT-8 cells (Figure 1). In contrast, PAR-2 mRNA was barely detectable by Northern blot analysis in other colon cancer cell lines including LS-174T, LoVo, SW620 and HCT116 (Figure 1). These data suggest that significant PAR-2 mRNA expression is detected in about 60% of colon cancer cell lines with this technique. RT-PCR experiments using conditions for maximal amplifications (see Materials and methods) revealed a single PCR product of the expected size (505 bp) in LS-174T, LoVo, SW620 and HCT116 cell lines (Figure 2), suggesting that they do express a very low level of mRNA.

Two observations indicated that the expression of PAR-2 mRNA is not correlated to the state of differentiation of colon cancer cells. First, PAR-2 mRNA was similarly detected by Northern blot in the undifferentiated parent HT-29 cells and in the polarized Cl.19A cells (Figure 1). Second, PAR-2 mRNA was expressed by both undifferentiated Caco-2 cells in exponential growth phase (not shown) and by postconfluent Caco-2 cells (Figure 1), which exhibit enterocyte-like differentiation (Laburthe et al, 1987; Zweibaum et al, 1991; Darmoul et al, 1992).

In order to provide further evidence for PAR-2 expression, indirect immunofluorescence studies were carried out on some human

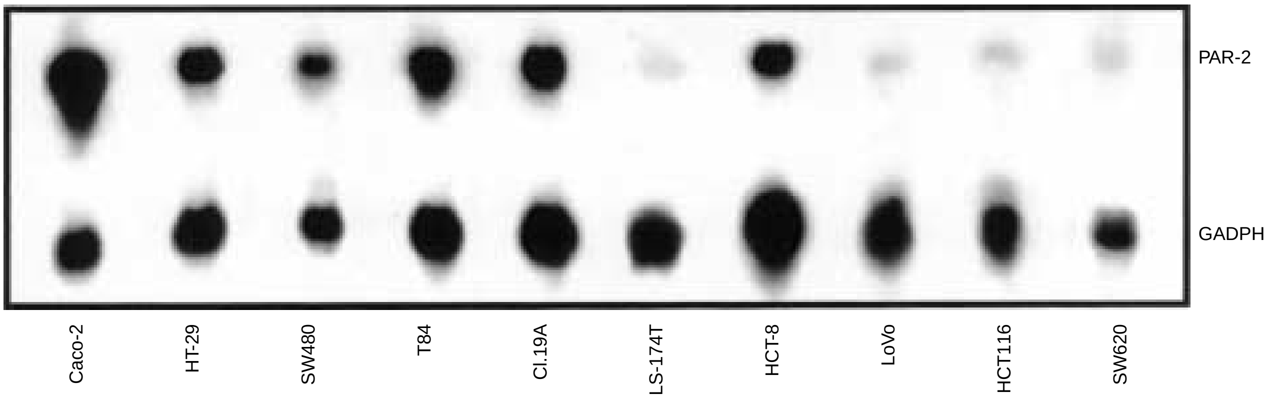

**Figure 1** Northern blot analysis of PAR-2 mRNA expression in various human colon cancer cell lines. Total RNA (10 μg/lane) was electrophoresed in formaldehyde-containing 1% agarose gel. Blots were first hybridized to human [$^{32}$P]-labelled PAR-2 cDNA probe. Blots were subsequently hybridized with [$^{32}$P]-labelled human GAPDH cDNA probe

 *© 2001 Cancer Research Campaign*

BJOC 01-1976 772-779 20/8/01 3:34 pm Page 775

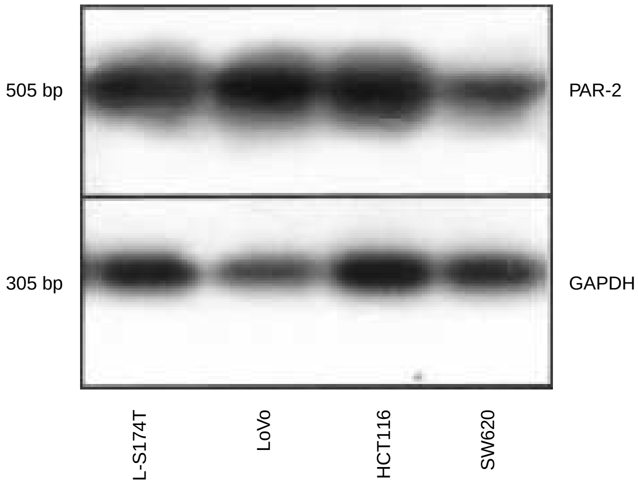

**Figure 2** PCR-based detection of PAR-2 mRNA in colon cancer cell lines. Total RNA extracted from various human colon cancer cells was reverse transcribed and PCR amplified with either PAR-2 or GAPDH primers. A single PCR-amplified product of the exact predicted size was visualized for both primer sets, after electrophoreses in a 2% agarose gel and hybridization with [$^{32}$P]-labelled full-length PAR-2 cDNA probe (upper panel) or [$^{32}$P]-labelled full-length GAPDH cDNA proble (lower panel)

colon cancer cell lines using the SAM11 monoclonal antibody to human PAR-2. Since this antibody recognizes an extracellular epitope located in the N-terminal domain of the receptor (Molino et al, 1998), studies were carried out on non-permeabilized cells. First, we investigated two cell lines exhibiting expression of PAR-2 mRNA in Northern blotting experiments, i.e. HT-29 and HCT-8. These studies allowed us to observe immunolocalization of PAR-2 on the cell surface of HT-29 and HCT-8 cells (Figure 3). No immunofluorescence was seen when only the second antibody was applied or when the primary antibody was pre-incubated with its immunizing peptide (not shown). Since PAR-2 has been shown to be internalized upon activation by trypsin or activating peptide AP2 (Bohm et al, 1996a), we also performed immunofluorescence studies following incubation of HT-29 cells with either activator. It appeared that cell surface labelling dramatically decreased (Figure 3), further assessing for the relevance of the immunofluorescence. Since some human colon cell lines appeared to express a low level of PAR-2 mRNA that could not be detected by Northern blotting (see above), an immunofluorescence experiment was also carried out on one such cell line, i.e. LS-174T. No fluorescence could be detected with LS-174T cells (Figure 3) suggesting a very low expression, if any, of the PAR-2 protein.

### PAR-2 activators and calcium mobilization

In order to characterize functional PAR-2 in human colon cancer cell lines, we first studied the role of PAR-2 activators on intra-cellular calcium mobilization in HT-29 cells expressing PAR-2 mRNA and PAR-2 protein at cell surface. Trypsin and the PAR-2 activating peptide AP2, corresponding to the neo-ligand formed

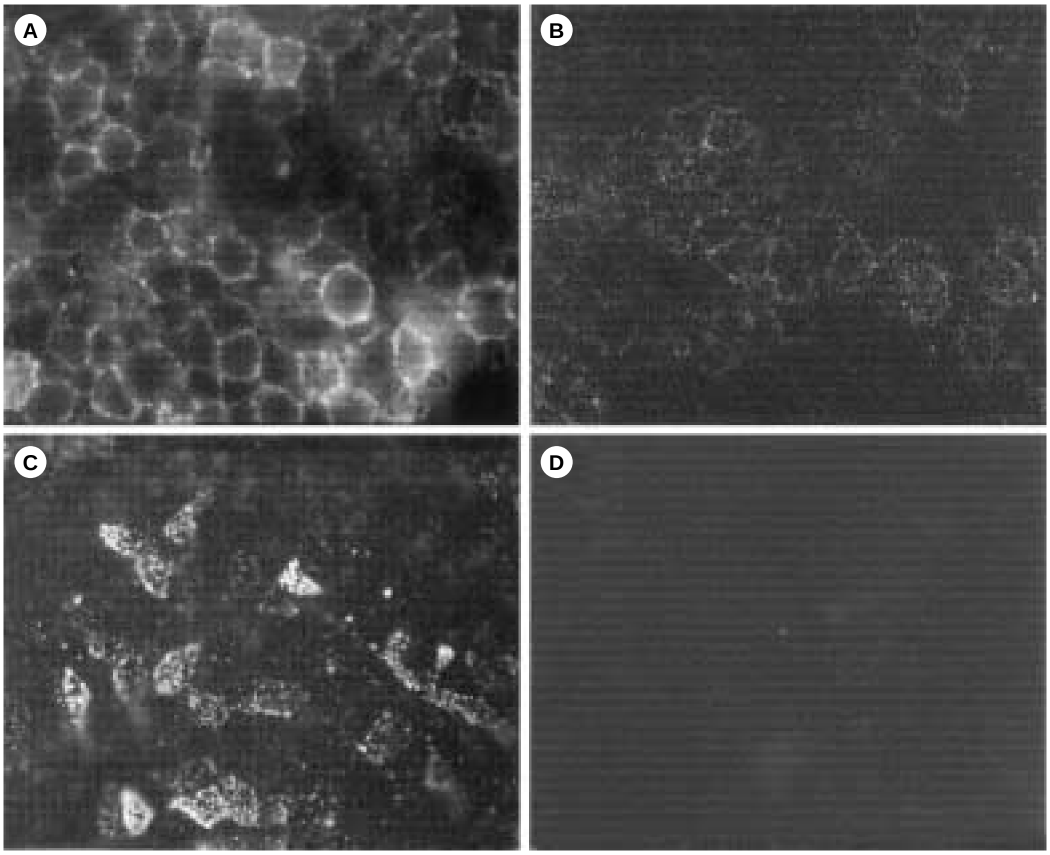

**Figure 3** Detection of PAR-2 in human colon cancer cells by immunofluorescence. Cells were fixed using 2% paraformaldehyde and immunostained with SAM11 monoclonal antibody as outlined in Materials and methods. (**A**) PAR-2 protein is seen on the cell surface of confluent HT-29 cells (400 ×). (**B**) HT-29 cells incubated at 37°C for 15 min with AP2 (100 μM) show very little immunofluorescence with SAM11 (400 ×). (**C**) Cell surface staining is evident in HCT-8 cells (400 ×). (**D**) No immunofluorescence was observed on LS-174T cell line (400 ×) stained with SAM11

© 2001 Cancer Research Campaign 

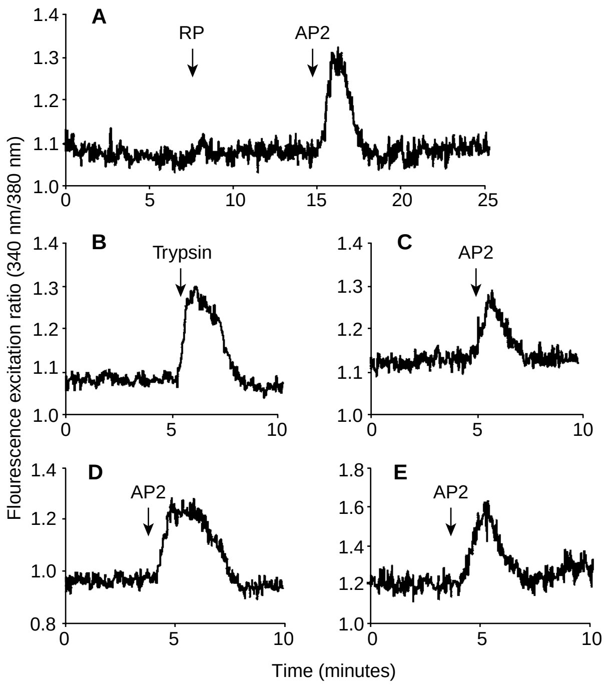

**Figure 4** Effect of trypsin and AP2 (SLIGKV) on calcium signalling. HT-29 cells were loaded for 60 min at 37°C using Fura-2/AM and assayed in medium containing $Ca^{2+}$ (**A, B**) or in $Ca^{2+}$-free medium (**C**). The addition of the activating peptide AP2 (100 μM), the inactive reverse peptide RP (100 μM) or trypsin (10 nM) is indicated by arrows. (**D**) and (**E**) show Cl.19A and HCT-8 cells challenged with 100 μM AP2, respectively. Representative experiments are shown. Each experiment has been carried out at least 3 times

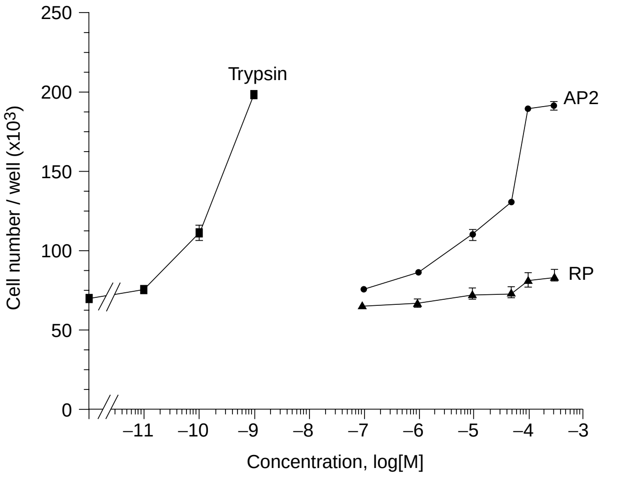

**Figure 5** Concentration-dependent stimulation of HT-29 cell proliferation by trypsin or AP2. Cells were seeded in medium containing 10% FCS. After 3 d, cells were washed and covered with serum-free medium for 48 h. Quiescent HT-29 cells were grown in serum free-medium with the indicated concentration of trypsin, synthetic PAR-2 agonist (AP2) or RP. After 96 h cells from triplicate wells were counted for each condition. Data are mean ± SEM

after receptor cleavage (SLIGKV), were used. As shown in Figure 4, activation of PAR-2 in HT-29 cells by 10 nM trypsin resulted in a rapid rise of intracellular calcium transients. After a first challenge by 10 nM trypsin followed by a 10-minute washing, a further challenge by 10 nM trypsin produced no calcium response (not shown). Although in our assay conditions it was difficult to determine dose-response curves, it is worth mentioning that 100 nM trypsin gave a response similar to that of 10 nM trypsin, while 1 nM enzyme only produced a small rise of intracellular calcium (not shown). The peptide AP2 also elicited a rapid rise of intracellular calcium (Figure 4). The peptide was active at 100 μM (Figure 4) or 50 μM, while 10 μM AP2 gave a small response (not shown). As a control it was verified that 100 μM reverse peptide (RP), whose sequence is VKGILS, had no effect on calcium mobilization in HT-29 cells (Figure 4). We also carried out experiments in which HT-29 cells were assayed in $Ca^{2+}$-free medium upon exposure to AP2 (100 μM) or trypsin (10 nM). The calcium responses to AP2 (Figure 4) or trypsin (not shown) were unaffected, indicating that $Ca^{2+}$ derives from intracellular pools.

The functional property of PAR-2 with respect to calcium mobilization was also studied in other human colon cancer cell lines exhibiting (Cl.19A, HCT-8) or not (LS-174T) detectable mRNA levels in Northern blot experiments (Figure 1). The peptide AP2 (100 μM) elicited a rapid rise of intracellular calcium transients in Cl.19A and HCT-8 cells (Figure 4). In contrast, no response could be observed in LS-174T cells (not shown). Neither was there any calcium mobilization in LS-174T cells upon challenge with 10 nM or 100 nM trypsin (not shown).

### PAR-2 activators and cell proliferation

The potential role of PAR-2 activators on proliferation of human colon cancer cells was first investigated on HT-29 cells. In the proliferation assay conditions used (see Materials and methods), 0.1 nM trypsin elicited a highly significant effect on cell proliferation (Figure 5). A maximal 300% increase of cell number above basal level was observed upon challenge of cells with 1 nM trypsin. After incubation with 0.1–1 nM trypsin, cells showed similar morphology as compared to untreated cells (data not shown). At a higher enzyme concentration (10 nM), an important detachment of HT-29 cells from plastic wells occurred and the proliferation assay was no longer reliable. The peptide AP2 also elicited a dose-dependent mitogenic response on HT-29 cells (Figure 5). Maximal response was observed at 100–300 μM peptide and represented a 270% increase of cell number, i.e. the same maximal response as for trypsin (300%). Half-maximal response was obtained for 47 ± 6 μM ($n = 8$) AP2. Unlike trypsin, AP2 did not induce HT-29 cell detachment regardless of the used peptide concentration (not shown). Maximal responses elicited by trypsin or AP2 were very important since they represented 30% of the maximal mitogenic response induced by 10% FCS, which reached a maximum of ~10-fold above serum-starved controls (not shown). The RP had no effect on HT-29 cell proliferation when tested up to 300 μM (Figure 5).

Further studies were carried out in order to investigate the role of PAR-2 activators trypsin and AP2 on proliferation of other human colon cancer cell lines. As shown in Figure 6, trypsin elicited a significant increase of cell number for Cl.19A and HCT-8 cells though the mitogenic effect is less pronounced than for HT-29 cells. Trypsin (1 nM)-induced increases of cell number above basal level represented 231% and 162%, respectively. In sharp contrast, trypsin had no effect on proliferation of LS-174T cells, which do not express PAR-2 at cell surface (see earlier). Neither was there any effect of trypsin on proliferation of HCT116 cells (Figure 6) that have a low PAR-2 mRNA level (Figure 1). The

 *© 2001 Cancer Research Campaign*

BJOC 01-1976 772-779 20/8/01 3:34 pm Page 777

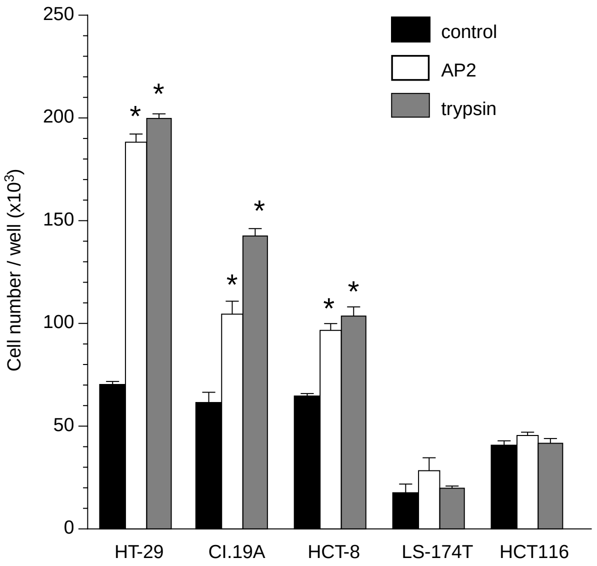

**Figure 6** Ability of PAR-2 ligands (AP2, trypsin) to induce mitogenesis in different human colon cancer cell lines. Cells were seeded in medium containing 10% FCS. After 3 d, cells were washed and covered with serum-free medium for 48 h. Quiescent human colon cancer cells were then grown for 96 h in serum free-medium without (control) or with 100 μM AP2 (AP2), or with 1 nM trypsin (Trypsin). Cells from triplicate wells were counted for each condition. Data are mean ± SEM. **P* < 0.001, Trypsin- or AP2-treated cells vs control cells (Student's *t*-test)

peptide AP2 gave responses that are similar to those triggered by trypsin. Indeed, AP2 stimulated proliferation of Cl.19A and HCT-8 cells but had no effect on proliferation of LS-174T and HCT116 cells (Figure 6).

### DISCUSSION

We have found that trypsin strongly stimulates proliferation of human colon cancer cells in culture. The mitogenic effect is promoted by low nanomolar concentration of trypsin and is mediated by a G protein-coupled seven-transmembrane domain receptor, PAR-2. Since PAR-2 expression and mitogenic effect of trypsin are observed in many colon cancer cell lines, we speculate that trypsin, and possibly other serine proteases acting at PAR-2, could represent new important signalling proteins in the control of colon cancer growth.

Northern blot analysis showed that PAR-2 mRNA is detectable in 60% of human colon cancer cell lines examined, e.g. HT-29, Caco-2, T84, Cl.19A, SW480 and HCT-8. Moreover, RT-PCR analysis provided evidence for the presence of PAR-2 transcripts in the remaining cell lines, suggesting that they express low levels of PAR-2 mRNA. A previous report also described PAR-2 transcripts in some colon cancer cell lines (Bohm et al, 1996b) but did not assess PAR-2 protein expression and function. In this study, immunofluorescence experiments showed that PAR-2 protein is expressed in cell lines exhibiting abundant transcripts such as HT-29 or HCT-8, whereas no staining could be observed with the anti-PAR-2 antibodies in cell lines with low expression of transcripts. The correlation extends to PAR-2 function since trypsin or the synthetic agonist AP2 promoted calcium mobilization and cell proliferation in cell lines expressing abundant PAR-2 mRNA and proteins such as HT-29 or HCT-8 but not in cell lines with a low number of transcripts or no staining in immunofluorescence studies such as LS-174T or HCT116. Altogether, our data support the idea that abundant mRNAs trigger the synthesis of functional PAR-2 protein in many, but not all, colon cancer cell lines. The expression of abundant PAR-2 mRNA or PAR-2 protein in colon cancer cells is not correlated with the differentiation phenotype of cells in culture. Indeed, PAR-2 is detected in cells that undergo enterocyte like-differentiation such as Caco-2 (Laburthe et al, 1987; Darmoul et al, 1992) or Cl.19A cells (Augeron and Laboisse, 1984) and also in cells that do not exhibit enterocyte differentiation markers (Zweibaum et al, 1991; Darmoul et al, 1992).

Proliferation of human colon cancer cells is stimulated by trypsin at very low concentrations in the 0.1–1 nM range (see Figure 5). These concentrations are much lower than the concentrations of trypsin required for hydrolysis of proteins in the duodenal lumen (Green and Nasset, 1980). Further, they are lower than the concentrations of trypsin required to trigger PAR-2-mediated regulation of short-term events such as prostaglandin secretion in small intestine enterocytes (Kong et al, 1997) or net electrogenic ion transport in pancreatic duct epithelial cells (Nguyen et al, 1999) that are in the micromolar range. This suggests that colonic cancer cells may have an exquisite sensitivity to trypsin. However, the effects of trypsin on colonic cancer cell proliferation are clearly mediated by PAR-2 since we showed that the specific agonist of PAR-2, AP2 (Blackhart et al, 1996; Hollenberg et al, 1997) also promoted cell proliferation, mimicking the trypsin effect. In contrast the reverse peptide with the reverse AP2 peptide sequence was devoid of any mitogenic effect. The same held true for control of calcium mobilization in colon cancer cells with AP2 mimicking trypsin effect and the reverse peptide being inactive (see Figure 4). As previously reported by others (see Vergnolle, 2000 for a review) AP2 is a low potency agonist compared to trypsin. The differences in the potency of AP2 are probably due to the non-sufficient presentation of AP2 to the binding domain of PAR-2. The same anchored amino acid sequence may be presented in a better conformation to the binding domain during the physiological activation of PAR-2 by trypsin.

Altogether, our data are consistent with a PAR-2-mediated control of colon cancer cell proliferation by trypsin. Trypsin or AP2 were very efficient in promoting colon cancer cell proliferation since, at maximally active concentrations, their effect represented up to a 300% increase of cell number in HT-29 cells. The effect of trypsin even represented 30% of the maximal mitogenic response induced by 10% FCS in HT-29 cells. Considering the high efficacy and potency of trypsin in promoting colon cancer cell proliferation, this serine protease and possibly other serine proteases acting at PAR-2 (see later), should be considered as growth factors that could be as significant as classical growth factors (Singh and Rubin, 1993; Normanno et al, 1998) or peptide hormones (Maoret et al, 1999; Singh et al, 2000) for colon cancer. Although the intracellular pathways responsible for PAR-2-mediated stimulation of colon cancer cell proliferation by trypsin are beyond the scope of this paper, it is worth noting that activation of PAR-2 results in PKC-dependent activation of MAP kinase cascade (Belham et al, 1996; DeFea et al, 2000) and phosphorylation of Src homology-2 domain-containing protein-tyrosine phosphatase SHP2 (Yu et al, 1997). These events are consistent with the PAR-2-mediated mitogenic responses that were observed in our studies.

The efficient and potent mitogenic action of trypsin on colon cancer cells raises the question of the endogenous source(s) of trypsin or other serine proteases that could activate PAR-2 in situ

*© 2001 Cancer Research Campaign* 

in colon cancer. Although low trypsin activity originating from exocrine pancreas may remain in the luminal content of the colon (Bustos et al, 1994), it is by far lower than the luminal trypsin content of the upper small intestine. In this context, it is worth pointing out that several lines of evidence indicate the importance of locally secreted trypsin at the vicinity of colon tumours: (1) trypsin was shown to be expressed by the large intestine (Koshikawa et al, 1998). Normal epithelial cells surrounding colon tumour cells are a likely source of active trypsin; (2) human colon cancer cells themselves were shown to express trypsin and trypsinogen (Bernard-Perrone et al, 1998; Miyata et al, 1999); (3) recent studies on gastric cancer indicate that blood vessels surrounding tumours express trypsin, whereas other blood vessels do not (Koshikawa et al, 1997). Trypsin is also present in serum at nanomolar concentrations and could diffuse from blood to tumour cells. Moreover, elevated trypsin levels were reported in the serum of patients with gastric carcinoma (Ichikawa et al, 2000), suggesting that serum may be an important source of trypsin in cancerous patients. Finally, many studies showed that tryptase originating from mast cells, also activates PAR-2 (Mirza et al, 1997; Molino et al, 1997a; Akers et al, 2000) though with a reduced potency and efficacy as compared to trypsin (Molino et al, 1997a). Since mast cells are abundant in colonic mucosa (Aldenborg and Enerback, 1994) and have been found to infiltrate colon tumour, it can be suggested that tryptase is a putative activator of PAR-2 in colonic tumours. In our recent studies, we showed that tryptase was also able to promote colonic cancer cell proliferation (Darmoul et al, unpublished results). Since the initial characterization of PAR-2 and its activation by trypsin, other activating serine proteases have been reported. These include tryptase, the membrane-type serine proteaseI, the tissue factor VIIa Xa complex (see O'Brien et al, 2001 for a review), a proteinase isolated from *Porphyromonas gingivalis* (Vergnolle, 2000) and acrosin (Smith et al, 2000). Among all these serine proteases, only tryptase and the tissue factor VIIa Xa complex might reasonably be expected to be present within the colonic cancer environment. Whether these proteases act on colonic cancer cell proliferation remains to be demonstrated.

In conclusion, we have shown the expression of functional PAR-2 in human colon cancer cells and demonstrated that trypsin has a potent and efficient mitogenic effect on colon cancer cells. These data support the hypothesis that trypsin can play an important role as a signalling protein in the growth of tumour of the colon.

### ACKNOWLEDGEMENTS

The authors thank Dr Brass for providing the PAR-2 antibody SAM11 and Dr Sundelin for the PAR-2 cDNA. We also thank Pascal Nicole and Jean-José Maoret for technical assistance with computer analysis and fluorescence imaging, respectively.

### REFERENCES

Akers IA, Parsons M, Hill MR, Hollenberg MD, Sanjar S, Laurent GJ and McAnulty RJ (2000) Mast cell tryptase stimulates human lung fibroblast proliferation via protease-activated receptor-2. *Am J Physiol Lung Cell Mol Physiol* **278**: L193–201

Aldenborg F and Enerback L (1994) The immunohistochemical demonstration of chymase and tryptase in human intestinal mast cells. *Histochem J* **26**: 587–596

Augeron C and Laboisse CL (1984) Emergence of permanently differentiated cell clones in a human colonic cancer cell line in culture after treatment with sodium butyrate. *Cancer Res* **44**: 3961–3969

Belham CM, Tate RJ, Scott PH, Pemberton AD, Miller HR, Wadsworth RM, Gould GW and Plevin R (1996) Trypsin stimulates proteinase-activated receptor-2-dependent and -independent activation of mitogen-activated protein kinases. *Biochem J* **320**: 939–946

Bernard-Perrone F, Carrere J, Renaud W, Moriscot C, Thoreux K, Bernard P, Servin A, Balas D and Senegas-Balas F (1998) Pancreatic trypsinogen I expression during cell growth and differentiation of two human colon carcinoma cells. *Am J Physiol* **274**: G1077–1086

Blackhart BD, Emilsson K, Nguyen D, Teng W, Martelli AJ, Nystedt S, Sundelin J and Scarborough RM (1996) Ligand cross-reactivity within the protease-activated receptor family. *J Biol Chem* **271**: 16466–16471

Blasi F and Stoppelli MP (1999) Proteases and cancer invasion: from belief to certainty. AACR meeting on proteases and protease inhibitors in cancer, Nyborg, Denmark, 14–18 June 1998. *Biochim Biophys Acta* **1423**: R35–R44

Blumberg PM and Robbins PW (1975) Effect of proteases on activation of resting chick embryo fibroblasts and on cell surface proteins. *Cell* **6**: 137–147

Bohm SK, Khitin LM, Grady EF, Aponte G, Payan DG and Bunnett NW (1996a) Mechanisms of desensitization and resensitization of proteinase-activated receptor-2. *J Biol Chem* **271**: 22003–22016

Bohm SK, Kong W, Bromme D, Smeekens SP, Anderson DC, Connolly A, Kahn M, Nelken NA, Coughlin SR, Payan DG and Bunnett NW (1996b) Molecular cloning, expression and potential functions of the human proteinase-activated receptor-2. *Biochem J* **314**: 1009–1016

Bustos D, Tiscornia O, Caldarini MI, Negri G, Pons S, Ogawa K and De Paula JA (1994) Colonic proteolysis following pancreatic duct ligation in the rat. *Int J Pancreatol* **16**: 45–49

Carney DH and Cunningham DD (1977) Initiation of check cell division by trypsin action at the cell surface. *Nature* **268**: 602–606

Chirgwin JM, Przybyla AE, MacDonald RJ and Rutter WJ (1979) Isolation of biologically active ribonucleic acid from sources enriched in ribonuclease. *Biochemistry* **18**: 5294–5299

D'Andrea MR, Derian CK, Leturcq D, Baker SM, Brunmark A, Ling P, Darrow AL, Santulli RJ, Brass LF and Andrade-Gordon P (1998) Characterization of protease-activated receptor-2 immunoreactivity in normal human tissues. *J Histochem Cytochem* **46**: 157–164

Darmoul D, Lacasa M, Baricault L, Marguet D, Sapin C, Trotot P, Barbat A and Trugnan G (1992) Dipeptidyl peptidase IV (CD 26) gene expression in enterocyte-like colon cancer cell lines HT-29 and Caco-2. Cloning of the complete human coding sequence and changes of dipeptidyl peptidase IV mRNA levels during cell differentiation. *J Biol Chem* **267**: 4824–4833

DeFea KA, Zalevsky J, Thoma MS, Dery O, Mullins RD and Bunnett NW (2000) beta-arrestin-dependent endocytosis of proteinase-activated receptor 2 is required for intracellular targeting of activated ERK1/2. *J Cell Biol* **148**: 1267–1281

Dery O, Corvera CU, Steinhoff M and Bunnett NW (1998) Proteinase-activated receptors: novel mechanisms of signaling by serine proteases. *Am J Physiol* **274**: C1429–1452

Green GM and Nasset ES (1980) Importance of bile in regulation of intraluminal proteolytic enzyme activities in the rat. *Gastroenterology* **79**: 695–702

Hedstrom J, Haglund C, Haapiainen R and Stenman UH (1996) Serum trypsinogen-2 and trypsin-2-alpha(1)-antitrypsin complex in malignant and benign digestive-tract diseases. Preferential elevation in patients with cholangiocarcinomas. *Int J Cancer* **66**: 326–331

Hirahara F, Miyagi Y, Miyagi E, Yasumitsu H, Koshikawa N, Nagashima Y, Kitamura H, Minaguchi H, Umeda M and Miyazaki K (1995) Trypsinogen expression in human ovarian carcinomas. *Int J Cancer* **63**: 176–181

Hollenberg MD (1996) Protease-mediated signalling: new paradigms for cell regulation and drug development. *Trends Pharmacol Sci* **17**: 3–6

Hollenberg MD, Saifeddine M, al-Ani B and Kawabata A (1997) Proteinase-activated receptors: structural requirements for activity, receptor cross-reactivity, and receptor selectivity of receptor-activating peptides. *Can J Physiol Pharmacol* **75**: 832–841

Ichikawa Y, Koshikawa N, Hasegawa S, Ishikawa T, Momiyama N, Kunizaki C, Takahashi M, Moriwaki Y, Akiyama H, Yamaoka H, Yanoma S, Tsuburaya A, Nagashima Y, Shimada H and Miyazaki K (2000) Marked increase of trypsin(ogen) in serum of linitis plastica (gastric cancer, borrmann 4) patients [In Process Citation]. *Clin Cancer Res* **6**: 1385–1388

Ishihara H, Connolly AJ, Zeng D, Kahn ML, Zheng YW, Timmons C, Tram T and Coughlin SR (1997) Protease-activated receptor 3 is a second thrombin receptor in humans. *Nature* **386**: 502–506

Kawano N, Osawa H, Ito T, Nagashima Y, Hirahara F, Inayama Y, Nakatani Y, Kimura S, Kitajima H, Koshikawa N, Miyazaki K and Kitamura H (1997) Expression of gelatinase A, tissue inhibitor of metalloproteinases-2, matrilysin, and trypsin(ogen) in lung neoplasms: an immunohistochemical study. *Hum*

*© 2001 Cancer Research Campaign*

BJOC 01-1976 772-779 20/8/01 3:34 pm Page 779

*Pathol* **28**: 613–622
Kennedy AR (1994) Prevention of carcinogenesis by protease inhibitors. *Cancer Res* **54**: 1999s–2005s
Kennedy AR (1998) The Bowman-Birk inhibitor from soybeans as an anticarcinogenic agent. *Am J Clin Nutr* **68**: 1406s–1412s
Kong W, McConalogue K, Khitin LM, Hollenberg MD, Payan DG, Bohm SK and Bunnett NW (1997) Luminal trypsin may regulate enterocytes through proteinase-activated receptor 2. *Proc Natl Acad Sci USA* **94**: 8884–8889
Koshikawa N, Hasegawa S, Nagashima Y, Mitsuhashi K, Tsubota Y, Miyata S, Miyagi Y, Yasumitsu H and Miyazaki K (1998) Expression of trypsin by epithelial cells of various tissues, leukocytes, and neurons in human and mouse. *Am J Pathol* **153**: 937–944
Koshikawa N, Nagashima Y, Miyagi Y, Mizushima H, Yanoma S, Yasumitsu H and Miyazaki K (1997) Expression of trypsin in vascular endothelial cells. *FEBS Lett* **409**: 442–448
Koshikawa N, Yasumitsu H, Umeda M and Miyazaki K (1992) Multiple secretion of matrix serine proteinases by human gastric carcinoma cell lines. *Cancer Res* **52**: 5046–5053
Laburthe M, Rousset M, Rouyer-Fessard C, Couvineau A, Chantret I, Chevalier G and Zweibaum A (1987) Development of vasoactive intestinal peptide-responsive adenylate cyclase during enterocytic differentiation of Caco-2 cells in culture. Evidence for an increased receptor level. *J Biol Chem* **262**: 10180–10184
Maoret JJ, Anini Y, Rouyer-Fessard C, Gully D and Laburthe M (1999) Neurotensin and a non-peptide neurotensin receptor antagonist control human colon cancer cell growth in cell culture and in cells xenografted into nude mice. *Int J Cancer* **80**: 448–454
Mazzieri R, Masiero L, Zanetta L, Monea S, Onisto M, Garbisa S and Mignatti P (1997) Control of type IV collagenase activity by components of the urokinase-plasmin system: a regulatory mechanism with cell-bound reactants. *EMBO J* **16**: 2319–2332
McCawley LJ and Matrisian LM (2000) Matrix metalloproteinases: multifunctional contributors to tumor progression. *Mol Med Today* **6**: 149–156
Mignatti P and Rifkin DB (1993) Biology and biochemistry of proteinases in tumor invasion. *Physiol Rev* **73**: 161–195
Mirza H, Schmidt VA, Derian CK, Jesty J and Bahou WF (1997) Mitogenic responses mediated through the proteinase-activated receptor-2 are induced by expressed forms of mast cell alpha-or beta-tryptases. *Blood* **90**: 3914–3922
Miyata S, Koshikawa N, Higashi S, Miyagi Y, Nagashima Y, Yanoma S, Kato Y, Yasumitsu H and Miyazaki K (1999) Expression of trypsin in human cancer cell lines and cancer tissues and its tight binding to soluble form of Alzheimer amyloid precursor protein in culture. *J Biochem (Tokyo)* **125**: 1067–1076
Miyata S, Miyagi Y, Koshikawa N, Nagashima Y, Kato Y, Yasumitsu H, Hirahara F, Misugi K and Miyazaki K (1998) Stimulation of cellular growth and adhesion to fibronectin and vitronectin in culture and tumorigenicity in nude mice by overexpression of trypsinogen in human gastric cancer cells. *Clin Exp Metastasis* **16**: 613–622
Molino M, Barnathan ES, Numerof R, Clark J, Dreyer M, Cumashi A, Hoxie JA, Schechter N, Woolkalis M and Brass LF (1997a) Interactions of mast cell tryptase with thrombin receptors and PAR-2. *J Biol Chem* **272**: 4043–4049
Molino M, Raghunath PN, Kuo A, Ahuja M, Hoxie JA, Brass LF and Barnathan ES (1998) Differential expression of functional protease-activated receptor-2 (PAR-2) in human vascular smooth muscle cells. *Arterioscler Thromb Vasc Biol* **18**: 825–832
Molino M, Woolkalis MJ, Reavey-Cantwell J, Pratico D, Andrade-Gordon P, Barnathan ES and Brass LF (1997b) Endothelial cell thrombin receptors and PAR-2. Two protease-activated receptors located in a single cellular environment. *J Biol Chem* **272**: 11133–11141
Nguyen TD, Moody MW, Steinhoff M, Okolo C, Koh DS and Bunnett NW (1999) Trypsin activates pancreatic duct epithelial cell ion channels through proteinase-activated receptor-2. *J Clin Invest* **103**: 261–269
Normanno N, De Luca A, Salomon DS and Ciardiello F (1998) Epidermal growth factor-related peptides as targets for experimental therapy of human colon carcinoma. *Cancer Detect Prev* **22**: 62–67
Nystedt S, Emilsson K, Larsson AK, Strombeck B and Sundelin J (1995) Molecular cloning and functional expression of the gene encoding the human proteinase-activated receptor 2. *Eur J Biochem* **232**: 84–89
Nystedt S, Emilsson K, Wahlestedt C and Sundelin J (1994) Molecular cloning of a potential proteinase activated receptor. *Proc Natl Acad Sci USA* **91**: 9208–9212
O’Brien PJ, Molino M, Kahn M and Brass LF (2001) Protease activated receptors: theme and variations. *Oncogene* **20**: 1570–1581
Ramos-DeSimone N, Hahn-Dantona E, Sipley J, Nagase H, French DL and Quigley JP (1999) Activation of matrix metalloproteinase-9 (MMP-9) via a converging plasmin/stromelysin-1 cascade enhances tumor cell invasion. *J Biol Chem* **274**: 13066–13076.
Saifeddine M, al-Ani B, Cheng CH, Wang L and Hollenberg MD (1996) Rat proteinase-activated receptor-2 (PAR-2): cDNA sequence and activity of receptor-derived peptides in gastric and vascular tissue. *Br J Pharmacol* **118**: 521–530
Singh P and Rubin N (1993) Insulinlike growth factors and binding proteins in colon cancer. *Gastroenterology* **105**: 1218–1237
Singh P, Velasco M, Given R, Wargovich M, Varro A and Wang TC (2000) Mice overexpressing progastrin are predisposed for developing aberrant colonic crypt foci in response to AOM. *Am J Physiol Gastrointest Liver Physiol* **278**: G390–G399
Smith R, Jenkins A, Lourbakos A, Thompson P, Ramakrishnan V, Tomlinson J, Deshpande U, Johnson DA, Jones R, Mackie EJ and Pike RN (2000) Evidence for the activation of PAR-2 by the sperm protease, acrosin: expression of the receptor on oocytes. *FEBS Lett* **484**: 285–290
Sorsa T, Salo T, Koivunen E, Tyynela J, Konttinen YT, Bergmann U, Tuuttila A, Niemi E, Teronen O, Heikkila P, Tschesche H, Leinonen J, Osman S and Stenman UH (1997) Activation of type IV procollagenases by human tumor-associated trypsin-2. *J Biol Chem* **272**: 21067–21074
Vergnolle N (2000) Review article: proteinase-activated receptors – novel signals for gastrointestinal pathophysiology. *Aliment Pharmacol Ther* **14**: 257–266
Vergnolle N, Macnaughton WK, Al-Ani B, Saifeddine M, Wallace JL and Hollenberg MD (1998) Proteinase-activated receptor 2 (PAR2)-activating peptides: identification of a receptor distinct from PAR2 that regulates intestinal transport. *Proc Natl Acad Sci USA* **95**: 7766–7771
Vu TK, Hung DT, Wheaton VI and Coughlin SR (1991) Molecular cloning of a functional thrombin receptor reveals a novel proteolytic mechanism of receptor activation. *Cell* **64**: 1057–1068
Xu WF, Andersen H, Whitmore TE, Presnell SR, Yee DP, Ching A, Gilbert T, Davie EW and Foster DC (1998) Cloning and characterization of human protease-activated receptor 4. *Proc Natl Acad Sci USA* **95**: 6642–6646
Yu Z, Ahmad S, Schwartz JL, Banville D and Shen SH (1997) Protein-tyrosine phosphatase SHP2 is positively linked to proteinase-activated receptor 2-mediated mitogenic pathway. *J Biol Chem* **272**: 7519–7524
Zweibaum A, Laburthe M, Grasset E and Louvard D (1991) The gastrointestinal system: Intestinal absorption and secretion. In: Field M and Frizzell RA (eds), Vol IV pp 223–255. American Physiological Society: Bethesda, MD, USA

© 2001 Cancer Research Campaign 
